# Robotic hormonal and autonomic modulation for type 2 diabetes: ileal interposition and hepatic sympathectomy

**DOI:** 10.3389/fsurg.2025.1657656

**Published:** 2025-12-09

**Authors:** Alcides Jose Branco Filho, Marcos Brioschi, Gabriel Carneiro Brioschi, Rafaella Monteiro Barbosa, Virgílio Frota Rossato, Luiz Alfredo Vieira d’Almeida

**Affiliations:** 1 Alcides Branco Medical Associates Clinic, Curitiba, Brazil; 2 Harvard T.H. Chan School of Public Health, Boston, MA, United States; 3 Faculty of Medicine, University of São Paulo (USP), São Paulo, Brazil; 4 Kent State University, Kent, OH, United States; 5 Pontifical Catholic University of Paraná, Curitiba, Brazil; 6 Red Cross Hospital, Curitiba, Brazil; 7 Obesity Treatment Center, Rio de Janeiro, Brazil

**Keywords:** type 2 diabetes, robotic surgery, ileal interposition, sympathectomy, metabolic surgery, perspective article

## Abstract

**Background:**

Type 2 diabetes mellitus (T2DM) is a complex disorder involving both hormonal imbalance and autonomic dysfunction. While bariatric surgery enhances glycemic control, its effects on autonomic regulation are limited.

**Objective:**

This is the first report describing two novel robotic surgical strategies targeting dual physiological mechanisms—hormonal modulation and sympathetic overactivity—to optimize metabolic outcomes in T2DM. The manuscript is intended to spark discussion around future surgical strategies based on two proof-of-concept index cases.

**Methods:**

We present two patients with poorly controlled T2DM. Case 1 underwent robotic ileal interposition with sleeve gastrectomy and duodenal exclusion, aiming to enhance incretin response and reduce glucagon levels. Case 2 underwent sleeve gastrectomy and duodenoileal interposition combined with selective hepatic sympathectomy, targeting sympathetic overactivity and autonomic dysfunction. Both procedures were performed using a robotic platform to enhance surgical precision.

**Results:**

Both procedures were completed successfully with minimal complications. The first patient showed significant weight loss and reduced insulin requirements after 12 months. The second patient reported resolution of sleep apnea symptoms and decreased paresthesia in the lower limbs, suggesting autonomic improvement.

**Conclusion:**

These cases highlight the safety, feasibility, and physiological rationale of combining robotic metabolic procedures with targeted autonomic modulation. This integrated approach may offer a promising pathway for treating complex metabolic disorders by addressing both hormonal and neural dysfunction. Further studies are warranted to validate these findings.

## 1 Introduction

Type 2 diabetes mellitus (T2DM) remains a global health challenge with rising incidence and significant healthcare costs. This condition is often accompanied by comorbidities such as hypertension, obesity, and cardiovascular disease (1). Although pharmacological treatments continue to evolve, surgical interventions have demonstrated superior metabolic control in selected patients. Robotic-assisted surgery offers enhanced precision, improved ergonomics, and superior visualization, facilitating complex metabolic procedures (2). Ileal interposition with sleeve gastrectomy and duodenal exclusion has shown promise in improving glycemic control, while sympathetic nervous system modulation via sympathectomy has been hypothesized to play a role in the autonomic regulation of metabolism. These procedures are distinct from standard metabolic surgeries such as SADI-S (single-anastomosis duodeno-ileal bypass with sleeve), RYGB (Roux-en-Y gastric bypass), and DJBL (duodenal-jejunal bypass liner), which remain the most widely reported approaches in current clinical practice. The present report proposes an alternative pathway, combining hormonal and autonomic modulation in a robotic setting. Importantly, the intent of this report was to explore metabolic and autonomic effects rather than body mass reduction alone.

Based on the anatomical feasibility and dual physiological rationale, the authors explore a novel robotic surgical strategy integrating ileal interposition and selective sympathectomy. These procedures aim to simultaneously address metabolic and autonomic dysfunction in patients with T2DM. To our knowledge, no prior case series has described these robotic techniques integration of both approaches addressing dual pathways in metabolic disease in such detail. Herein, we report two clinical cases that illustrate the potential of integrating robotic innovation in metabolic and autonomic surgical approaches and raise hypotheses for further investigation.

## 2 Case reports

### 2.1 Case 1: robotic ileal interposition with duodenal exclusion and sleeve gastrectomy

A 61-year-old male patient was diagnosed with type 2 diabetes mellitus in 2004, at which time metformin therapy was initiated. Insulin treatment was added in 2009. In 2021 the patient presented with dyslipidemia, systemic arterial hypertension and obesity. In the pre-operative period he was taking dapagliflozin, metformin, pioglitazone, rapid-acting insulin and losartan. His height was 1.68 m, weight 93.9 kg and body mass index (BMI) 33.3 kg/m^2^ (class I obesity).

He had occasional capillary glucose peaks up to 400 mg/dl, with a mean preoperative fasting glucose of 149 mg/dl and glycated hemoglobin of 7.3%, fasting glucose 149 mg/dl and insulin 11.7 µIU/mL.

Based on the persistent poor metabolic control, in 2021 ileal interposition with duodenal exclusion and vertical gastrectomy by a robotic approach was proposed, taking into account the preserved pancreatic reserve and aiming at glucagon blockade.

#### 2.1.1 Intra-operative course

The procedure was started with robotic vertical gastrectomy. The stomach was left with approximately 100 mL, and the staple line was oversewn. The duodenum was then excluded 4.0 cm from the pylorus with a stapler. At 30 cm from the ileocaecal valve, 1.6 m of terminal ileum was divided using a 45 mm stapler load (USA-Medtronic®). This ileum was interposed between the duodenum and the jejunum 50 cm from the Treitz angle. The ileum was interposed with an end-to-side anastomosis to the duodenum. The distal part of the interposed ileum was laterally anastomosed to the jejunum 50 cm from the Treitz angle. Finally, the defects were closed with simple stitches. The operation lasted 6 h, with a total blood loss of only 150 mL and no complications. Diet was started on the second postoperative day and hospital discharge occurred on the fifth day.

*Extended intraoperative details, including reconstruction steps, suture techniques and trocar positioning, are provided in* Supplementary Material.

#### 2.1.2 Immediate post-operative period

The patient reported pain in the immediate postoperative period and had difficulty adapting to the recommended diet during the 15 days after discharge, with episodes of pain, vomiting and dumping syndrome. He adhered to the diet and progressed without symptoms.

#### 2.1.3 Follow-up

Eleven months after surgery he was taking sertraline 20 mg and empagliflozin 20 mg. After one year of follow-up his weight was 68 kg, with a BMI of 24.09 kg/m^2^. His glycated haemoglobin was 6.4%, insulin 2.6 µIU/mL and estimated average glucose 137 mg/dl.

### 2.2 Case 2: robotic sleeve gastrectomy and duodenoileal interposition with hepatic selective sympathectomy

A 56-year-old male patient with a BMI of 39 kg/m^2^ (130 kg, 1.76 m) was diagnosed with type 2 diabetes mellitus approximately nine years ago and also had systemic arterial hypertension. He reports a pre-operative weight loss of 10 kg. His medications were NPH insulin 28 IU in the morning and 32 IU at night, metformin XR 500 mg (1-0-1), glibenclamide 5 mg (1-0-1) and enalapril 10 mg (1-0-1). He had no history of prior surgery. Medical history included diabetic neuropathy and a family history of metabolic disease.

Pre-operative laboratory tests showed negative anti-GAD and anti-islet antibodies, C-peptide 1.15 ng/mL and glycated haemoglobin 9.5%. Myocardial scintigraphy revealed mild, transient hypocapture at the apex of the left ventricle and an ejection fraction of 65%. Carotid Doppler identified moderate intima-media thickening.

The patient underwent, on 30 May 2025, a metabolic surgery for obesity consisting of vertical gastrectomy associated with duodeno-ileal anastomosis plus selective sympathectomy using robotics. The indication for this combination was glucagon blockade. This surgical association was chosen on the basis of a dual pathophysiological rationale. Ileal interposition anticipates contact of food with the terminal ileum, promoting early release of incretin hormones such as GLP-1 and PYY, which reduce glucagon secretion and increase insulin sensitivity, being especially useful in patients with type 2 diabetes and preserved pancreatic reserve (3, 4). Selective sympathectomy was performed to modulate the sympathetic hyperactivity often present in diabetics with autonomic dysfunction, improving peripheral perfusion, insulin sensitivity and blood-pressure parameters. Combining these two approaches aims to enhance metabolic and autonomic effects, based on pre-clinical evidence and isolated reports in the literature (5, 6), and provides an anatomically feasible model within the robotic platform, without the technical conflicts that more complex malabsorptive techniques (such as RYGB) could impose during access to the abdominal sympathetic plexus.

#### 2.2.1 Intra-operative course

The robotic procedure used the following ports: right hemiclavicular line, right periumbilical, left hemiclavicular and lateral at the level of the anterior-superior iliac crest. A 5 mm trocar was placed in the epigastrium for a liver retractor and a 12 mm trocar infra-umbilically on the right for stapler manipulation. Instruments used were Cadiere, bipolar, scissors, needle holder and vessel sealer, with bipolar energy.

The gastric approach began with dissection and division of all short vessels along the greater curvature up to the angle of His. Duodenal dissection then created a window 6 cm from the pylorus. A gastric sleeve was fashioned with 60 mm stapler loads along the entire body and fundus.

**Figure 1:**
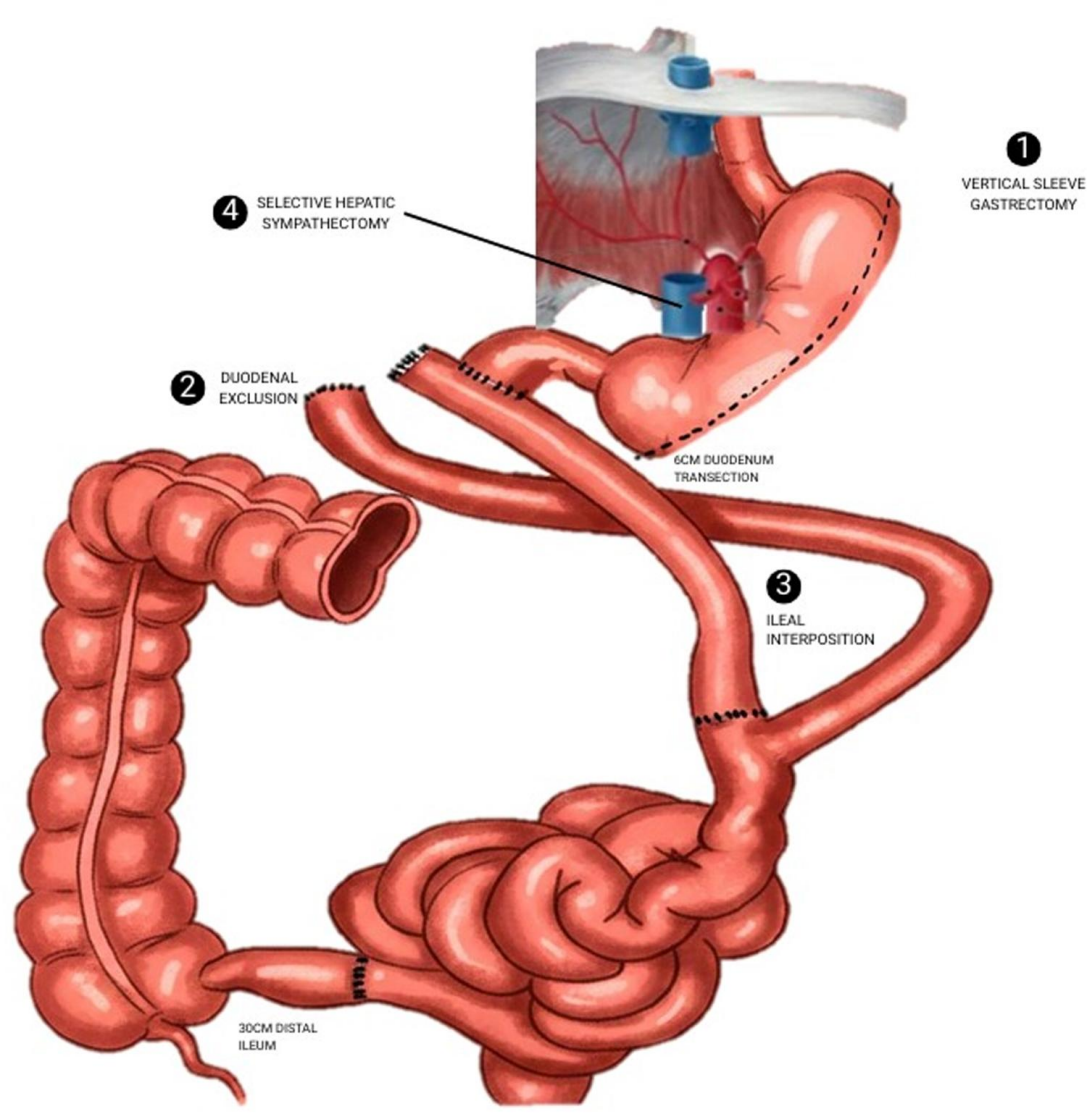
Schematic representation of the surgical strategy showing the postoperative configuration after vertical sleeve gastrectomy (1) with resection of the greater curvature and creation of a tubular gastric reservoir, duodenal exclusion (2) by transection of the duodenum 4–6 cm distal to the pylorus while preserving pyloric continuity, ileal interposition (3) with resection of a distal ileal segment 20–30 cm proximal to the ileocecal valve and its placement between the proximal duodenum and the jejunum 50 cm from the angle of Treitz to promote early nutrient exposure and incretin stimulation (GLP-1, PYY), and selective hepatic sympathectomy (4) performed by denervation of sympathetic fibers along the right diaphragmatic crus, periarterial plexus of the common hepatic artery, and adjacent to the inferior vena cava, thereby combining hormonal modulation and autonomic modulation to improve insulin sensitivity and metabolic control.

**Figure 2:**
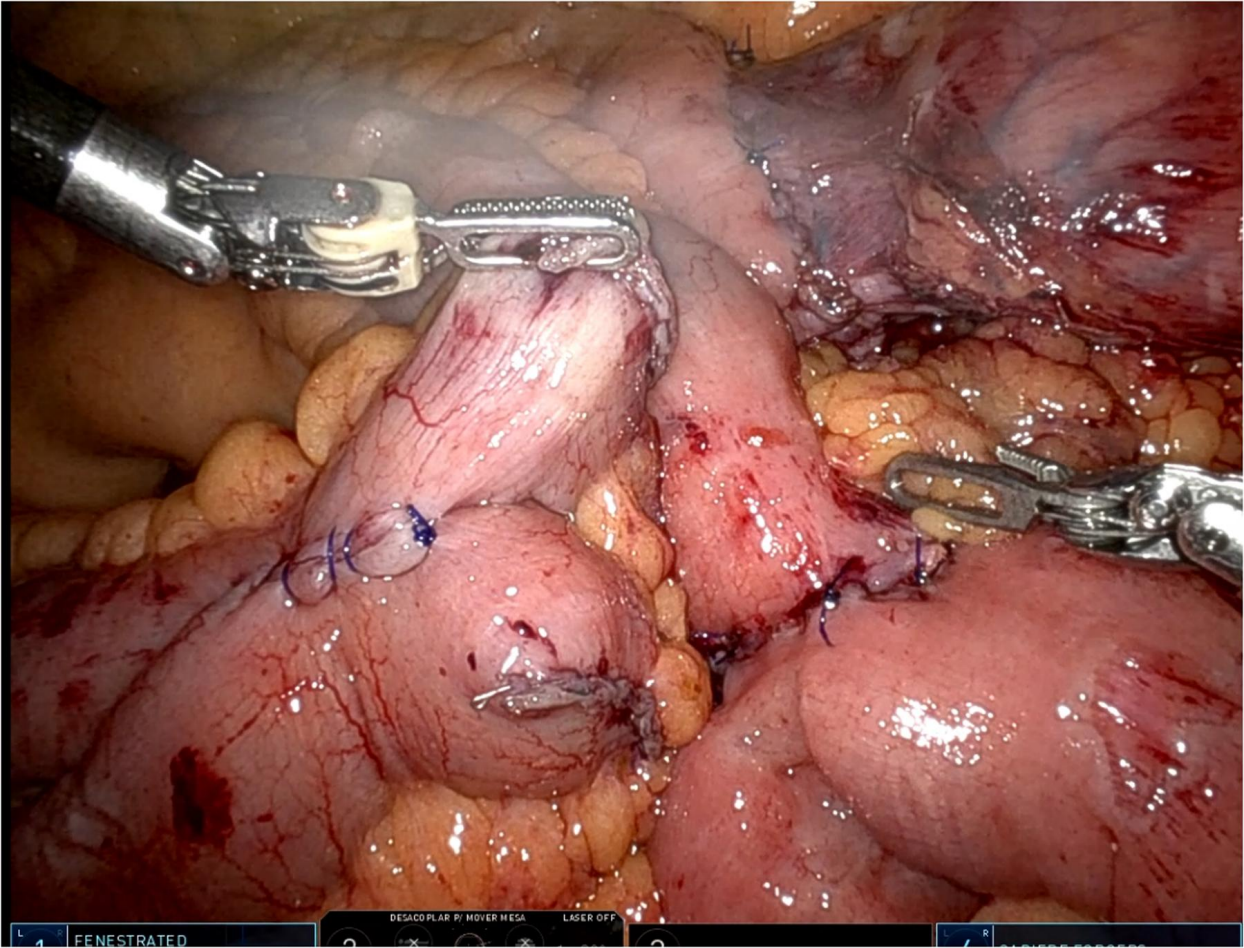
Intraoperative robotic view showing the anatomical configuration during the procedure with emphasis on the check of intestinal anastomoses. The robotic instruments are used to expose and test the anastomotic sites, confirming adequate vascularization and integrity of the sutures before completing the surgical step.

The duodenum was divided 6 cm from the pylorus using a 45 mm load. Right-crus sympathectomy was then performed at the cava, dividing two visible sympathetic nerves. Sympathectomy was carried out selectively around the common hepatic artery, just beyond the bifurcation of the gastroduodenal artery, with a 360-degree circumferential robotic dissection. This perivascular approach aims to interrupt afferent and efferent sympathetic conduction to the hepatic region, based on pathophysiological evidence linking hepatic sympathetic activity to insulin resistance and metabolic dysfunction in patients with type 2 diabetes (Figures 1, 2).

The greater omentum was subsequently mobilized. The distal ileum was divided 20 cm from the ileocecal valve. A forward count of 1.5 m from the distal ileal segment was measured and another division made with a 45 mm load.

A side-to-side ileo-ileal anastomosis was performed using a 45 mm load. Finally, a hand-sewn end-to-side ileo-jejunal anastomosis was created about 50 cm from the angle of Treitz, with a similar closure of the mesenteric defect.

The robotic procedure was completed in 270 min (console time ≈ 210 min). Estimated blood loss was <100 mL and no intra-operative complications—vascular injury, bile-duct lesion or need for conversion—were observed. Haemodynamic parameters remained stable throughout the operation and no vasoactive support was required, confirming the feasibility and safety of performing a 360-degree perivascular sympathectomy around the common hepatic artery within a single robotic session. No intra-operative incidents were observed.

Technical details of trocar configuration, sutures, and perivascular sympathectomy steps are described in Supplementary Material.

#### 2.2.2 Immediate post-operative period

The patient reported only mild pain (VAS 3/10) restricted to the port sites, controlled with dipyrone and paracetamol, with no need for opioids. He ambulated six hours after surgery and maintained haemodynamic stability, with no nausea or vomiting. After 12 h a high-protein liquid diet was started, with excellent tolerance and no signs of fistula, bleeding or fever in the first 24 h. **Case 2 was discharged on the 4th postoperative day, with no complications reported.**

At present he weighs 103 kg, with a weight loss of about 1 kg per week. No post-operative complications have been observed.

#### 2.2.3 Follow-up

The patient lost 1 kg per week with good control of capillary glucose, averaging 140 mg/dl. Current mean capillary glucose is 116 mg/dl and HbA1c 6.6%. Triglycerides fell to 49 mg/dl. Blood pressure stabilised at 130 × 80 mmHg with enalapril 10 mg/day. All insulin, metformin and glibenclamide were discontinued; gliclazide is used only if glucose >125 mg/dl. Although improvement in plantar paresthesia was observed, this effect is likely multifactorial and related to weight loss, improved glycemic control, and reduced systemic inflammation rather than a direct consequence of hepatic sympathectomy. The nutritionist documents full adherence to a soft high-protein diet and adequate hydration, with no gastrointestinal events.

In this second case, the patient reported improvement of symptoms often associated with autonomic dysfunction, such as reduced tingling in the feet, consistent with improvement of peripheral neuropathy of sympathetic pattern. In addition, the reported resolution of snoring during sleep may relate to improved autonomic respiratory regulation, as sympathectomy can impact upper-airway tone. Although a causal relationship between sympathectomy and these clinical improvements cannot yet be confirmed, these findings suggest possible beneficial effects on the peripheral sympathetic nervous system, consistent with the pathophysiological rationale for performing the procedure.

The patient followed a soft diet during the first post-operative month, with good acceptance and no relevant gastrointestinal symptoms. The diet consisted of easily digestible foods, prioritising high-biological-value proteins, low fat content and restriction of simple sugars. Despite the short follow-up, no diet-related clinical events occurred, and the patient maintained adequate adherence to the meal plan as recorded at follow-up visits. Laboratory tests 24 days after surgery showed glycated haemoglobin 6.6%, glucose 61 mg/dl and triglycerides 49 mg/dl, the latter having fallen by half.

## 3 Discussion

This study is not merely about anatomical reconstruction, but rather about the intentional dual mechanism that combines robotically assisted ileal interposition without duodenal exclusion—which preserves the pylorus and the native proximal intestinal continuity (unlike SADI-S)—with a selective robotic hepatic sympathectomy, which is not part of standard metabolic procedures such as SADI-S, RYGB, or even DJBL. To our knowledge, this combination had not previously been reported in humans, especially using robotic platforms with this level of precision. Our intention was not to present a new variation of SADI-S, but rather to highlight a proof-of-concept integrating endocrine and autonomic targets for type 2 diabetes and metabolic syndrome, with translational value derived from preclinical work.

Obesity has become a worldwide epidemic. Bariatric surgery has been considered an important treatment option for patients with severe obesity, that is, those with a body mass index (BMI) > 35 with at least one related comorbidity or BMI > 40 (2). According to the 2025 American Diabetes Association guidelines, metabolic surgery may also be considered in patients with BMI ≥ 30 kg/m^2^ when optimal glycemic control is not achieved with pharmacologic therapy (2).

Obesity and DM2 are characterized by hyperactivity of the sympathetic nervous system (SNS), which contributes to hypertension, insulin resistance and other metabolic dysfunctions.

However, the contribution of sympathetic overactivity to diabetes pathogenesis remains modest compared with its established role in hypertension. While experimental and limited clinical evidence suggest possible metabolic benefits from sympathetic denervation, the primary therapeutic impact of such interventions lies in symptomatic relief and improvement of vascular tone rather than direct glycemic control. The present concept therefore frames autonomic modulation as a complementary, not primary, mechanism within the broader metabolic treatment spectrum.

Metabolic surgery is a well-established treatment for type 2 diabetes mellitus (DM2) in obese patients, yet this fact raises the question of whether associated direct ablation of the sympathetic nervous system (sympathectomy) can be used to improve outcomes.

Laparoscopic surgery has been the gold-standard approach. Nonetheless recently, robotic surgery has emerged as a potential alternative, since it offers several potential advantages, including greater dexterity and precision in tissue manipulation (2, 7). Small blood loss and similar operative time have also been observed. These advantages—fine dissection, elimination of tremor and 3D visualisation—suggest that a robot could safely dissect autonomic nerve chains.

These cases were selected not merely for weight reduction but to investigate metabolic and autonomic pathways beyond BMI-driven indications. In patients with lower BMI, avoiding RYGB also reduces the risks of excessive weight loss and nutritional complications, while preserving the pylorus prevents severe dumping syndrome. Thus, the rationale for this approach was based on targeting both incretin pathways and sympathetic tone, rather than pursuing a purely restrictive or malabsorptive procedure. The choice of ileal interposition associated with vertical gastrectomy (instead of more widespread techniques such as RYGB or mini-gastric bypass) was based on three main reasons.

First, this technique preserves the pylorus and much of duodenal transit, minimizing the risk of dumping syndrome and alkaline gastritis, which are frequent in conventional bypasses. Unlike conventional procedures such as SADI-S, RYGB, or DJBL, the combination of ileal interposition with selective hepatic sympathectomy represents a novel dual-pathway strategy. This approach simultaneously targets hormonal incretin stimulation and autonomic modulation, providing a translational proof-of-concept that differs fundamentally from currently established metabolic operations.

Second, by transposing a distal ileal segment for early contact with nutrients, there is direct stimulation of GLP-1, PYY and other incretin peptide secretion, providing a powerful metabolic effect even in patients with moderate BMI and reducing the need for an aggressive malabsorptive component (8–12).

While incretin stimulation is also observed after sleeve gastrectomy and RYGB (7), human series of ileal interposition have demonstrated robust incretin effects and clinically meaningful glycemic improvements by De Paula et al. (3) and Cummings et al. (13). For context on durable metabolic efficacy of alternative disabsorptive procedures, long-term BPD data from Scopinaro's group show high rates of diabetes remission (14). By contrast, long-term follow-up of gastric bypass trials, including the STAMPEDE trial by Schauer et al. (15, 16), has shown remission rates declining to below 30% at five years. This evidence supports a translational rationale for investigating ileal interposition as an alternative pathway with potentially superior hormonal modulation and lower anatomical burden compared to standard bypass procedures.

Human data from the Swedish Obese Subjects trial demonstrates remission rates of type 2 diabetes of approximately 72% at 2 years with metabolic surgery, significantly higher than medical therapy alone (7, 16). Animal studies further support the mechanism of increased incretin secretion following ileal interposition with improved insulin sensitivity (13). These findings strengthen the rationale for selecting ileal interposition in our cases.

Third, the preserved anatomy facilitates the performance of selective robotic sympathectomy (crura and hepatic artery), because it avoids long biliopancreatic limbs and multiple jejunal staplings, allowing more direct access to the target nerve chains.

We recognise, however, that we did not directly compare this approach with established metabolic procedures (RYGB or mini-bypass). The absence of a comparative group represents an additional limitation of this study and prevents extrapolations about superiority or equivalence of the technique in relation to conventional options.

Despite the lack of clinical precedents, there is strong physiological grounding—supported by animal studies and some human data—that interruption of sympathetic nerves can modulate metabolic function in diabetes. The sympathetic nervous system influences glucose metabolism in multiple organs. SNS hyperactivity is a feature of metabolic syndrome and DM2, contributing to insulin resistance. Conversely, reduction of sympathetic tone can enhance insulin action and glucose uptake (8–12).

While robotic platforms are increasingly used in metabolic surgery, clinical outcomes of sympathectomy (surgical or endovascular) have shown promising effects in controlling glucose, insulin sensitivity and in reducing sympathetic overactivity in patients with T2DM or metabolic syndrome (13, 15).

Studies have shown that renal sympathetic denervation appears to significantly improve glucose metabolism in the short term, such as fasting blood glucose control, insulin secretion, C-peptide levels, insulin sensitivity, and glucose tolerance within 3 months, in patients with resistant hypertension (11, 12).

Experimental evidence supports the metabolic rationale for hepatic or lumbar sympathectomy in T2DM. In fat- and fructose-fed dogs, Kraft et al. demonstrated that selective denervation of the common hepatic artery improved glucose tolerance and reduced insulin resistance (8). Rodent studies by Pérez-Juárez et al. confirmed that lumbar sympathectomy lowered intra-abdominal catecholamine levels and enhanced peripheral glucose utilisation in streptozotocin-induced diabetes (9). Translational work has reached early clinical application: Pan et al. reported improved HbA1c and fasting glucose after endovascular celiac (hepatic) denervation in patients with uncontrolled T2DM (10), while Mahfoud et al. observed better insulin sensitivity following renal–sympathetic denervation in resistant hypertension (11). Collectively, these data suggest that targeted interruption of sympathetic efferents can modulate hepatic glucose output and peripheral insulin action, providing biological plausibility for combining perivascular sympathectomy with incretin-based surgical procedures. This report should therefore be interpreted as a translational proof-of-concept. While previous animal and early human studies have not replicated exactly the combination of ileal interposition with selective sympathectomy, the integration of endocrine and autonomic targets is grounded in current pathophysiological knowledge of T2DM. The intent was to generate hypotheses for future prospective trials rather than to claim superiority over established techniques at this stage.

Recent systematic reviews and translational studies highlight robotic platforms as advantageous in complex metabolic procedures, and sympathectomy—both surgical and endovascular—has shown promising clinical impact on glycemic control (10, 11).

This paper reports two cases of male patients with obesity and DM2 who underwent ileal interposition with vertical gastrectomy. In this surgery, duodenal exclusion is performed about 6 cm from the pylorus followed by interposition of ileum obtained about 20 cm from the ileocaecal valve and 1.50 m distal to this part, interposed between the duodenum and approximately 50 cm from the angle of Treitz. In the second case the surgical procedure included sympathectomy. This was carried out by approaching a nerve bundle near the right diaphragmatic crus next to the cava and another near the right hepatic artery with its isolation.

Although only one of the reported cases included a sympathectomy, the first case demonstrates the technical feasibility and metabolic potential of a complex robotic procedure combining ileal interposition, duodenal exclusion, and sleeve gastrectomy. This hormonal approach complements the autonomic modulation seen in the second case and reinforces the versatility of robotic surgery in addressing different metabolic pathways in T2DM.

The first case had 1-year follow-up and the second 1 month, which made comparison between them difficult. Nevertheless, in both cases there was a relevant reduction in body weight and improvement of DM2, with the improvement appearing quite rapid in the second case. In both cases robotic surgery provided greater comfort to the surgical team, as well as precision of movements, the main reason for choosing this route due to the crucial part of the sympathectomy. Operative time and blood loss were also similar to videolaparoscopic surgery (Table 1).

**Table 1:** Comparison of the main parameters and outcomes between the two presented robotic surgery cases.

Parameter	Case 1	Case 2
Age/Sex	61/Male	56/Male
Pre-operative BMI	33.3 kg/m^2^	39 kg/m^2^
Duration of DM2	12 years	9 years
Hypertension	Yes	Yes
Others comorbidities	No	No
Neuropathy/Autonomic dysfunction	No	Yes
Previous surgeries	No	No
Surgical procedure	Ileal interposition + duodenal exclusion + sleeve gastrectomy	Ileal interposition + duodenal exclusion + sleeve gastrectomy + sympathectomy
Robotic platform used	Da Vinci Xi (Strattner®))	Da Vinci X (Strattner®))
Sympathectomy details	Not performed	Splenic level
Operative time	6 h	4 h e 30 min
Estimated blood loss	150 ml	100 ml
Intra-operative complications	No	No
Post-operative pain	Immediate and after diet introduction	Immediate
Length of hospital stay	Five	Two
Diet progression	Well tolerated	Well tolerated
Follow-up duration	1 year	1 month
Weight loss and BMI change	68 kg and 24.09 kg/m	130 Kg and 33.2 kg/m^2^
Pre-op HbA1c	7.3%	9.5%
Post-op HbA1c	6.4%	6.6%
Reduction in antidiabetic medication	Empagliflozin use	Gliclazide use
Blood pressure evolution	Similar	Similar
Neuropathy/Autonomic changes	None	Symptom improvement
Patient-reported outcomes	Initial feeding difficulty but improved weight and laboratory results	Initial feeding difficulty but improved weight and laboratory results

Thus, robotic surgery is being increasingly used in bariatric/metabolic surgery, including in Brazil. To the best of our knowledge, this is the first case report describing the simultaneous association between robotic sympathectomy and metabolic surgery with ileal interposition in humans (Pubmed/Medline Database, BVS). This approach may represent a new frontier in the neuro-endocrine modulation of DM2.

Although this report provides important insights into the combination of sympathectomy and metabolic surgery using a robotic approach, it is important to acknowledge the limitations of this study. This article presents only two clinical cases, which inherently restricts the generalizability of the results. Case 2's short-term data represents preliminary feasibility. The asymmetry in follow-up is clearly acknowledged, but the comparison between cases is framed as illustrative, not comparative. The observed improvements may be influenced by individual variability, medication changes, and postoperative care, including dietary factors. Therefore, the findings should be interpreted with caution, and further research with larger case series or prospective studies is required to confirm the potential benefits and reproducibility of this combined technique.

This study represents a translational adaptation of prior experimental work. While no previous report has replicated exactly the combination of ileal interposition and selective sympathectomy, the integration of endocrine and autonomic targets is grounded in the current understanding of T2DM pathophysiology and aims to generate hypotheses for future clinical trials.

## 4 Conclusion

These cases demonstrate the feasibility and potential metabolic and autonomic benefits of combining robotic ileal interposition or sleeve gastrectomy with selective sympathetic denervation. And suggest that robotic sympathectomy may represent a novel adjunct to bariatric-metabolic surgery in selected T2DM patients with autonomic dysfunction. Robotic platforms provide the necessary precision, ergonomics, and dexterity to enable complex anatomical dissections—such as circumferential hepatic artery sympathectomy—in a minimally invasive manner in combination with tailored metabolic reconstructions. The observed metabolic improvements, including reduced medication requirements and glycemic control, along with signs suggestive of autonomic modulation (e.g., neuropathic symptom relief, improved blood pressure), highlight the potential benefit of this dual-modality surgical strategy. Further studies are warranted to validate these findings, elucidate long-term outcomes, and clarify the role of autonomic modulation in enhancing the efficacy of metabolic procedures.

## Data Availability

The original contributions presented in the study are included in the article/Supplementary Material, further inquiries can be directed to the corresponding author.
