# Supplementary material for: Robotic hormonal and autonomic modulation for type 2 diabetes: ileal interposition and hepatic sympathectomy
Source: Front Surg. 2025 Dec 9;12:1657656. doi: 10.3389/fsurg.2025.1657656 (PMC12722888; doi:10.3389/fsurg.2025.1657656)
Supplement: Supplementary file 1 [file Datasheet1.pdf]

Supplementary Material – Complete Operative Protocol
Case 1: Robotic Ileal Interposition with Duodenal Exclusion and Sleeve Gastrectomy

Trocar positioning and access
Standard four-port robotic configuration was used, including supra- and infra-umbilical access and auxiliary laparoscopic ports.
Gastric phase
- Vertical sleeve gastrectomy performed, leaving a gastric reservoir of ~100 mL.
- Staple line reinforced with continuous 3-0 PDS sutures.

Duodenal exclusion
- Division of duodenum 4 cm from the pylorus with a stapler.
- Oversewn with continuous 3-0 PDS.

Ileal interposition
- 1.6 m of terminal ileum isolated, 30 cm proximal to ileocecal valve.
- Ileum transposed between duodenum and jejunum 50 cm from the angle of Treitz.
- End-to-side duodeno-ileal anastomosis with continuous absorbable monofilament 3-0.
- Side-to-side ileo-ileal anastomosis with absorbable 3-0; defects closed with non-absorbable 3-0.
- Mesenteric gaps closed with non-absorbable 3-0.

Intra-operative outcomes
- Duration: 6 hours.
- Blood loss: 150 mL.
- No intra-operative complications.

Postoperative course
- Diet initiated POD 2.
- Hospital discharge POD 5.

Case 2: Robotic Sleeve Gastrectomy with Duodeno-Ileal Interposition and Hepatic Sympathectomy
Trocar positioning and access
- Robotic ports: right hemiclavicular, left hemiclavicular, right infra-umbilical, left lateral.
- 5 mm epigastric port for liver retractor; 12 mm infra-umbilical for stapler.
- Instruments: Cadiere, bipolar, scissors, needle holder, vessel sealer.

Gastric phase
- Division of short gastric vessels to the angle of His.
- Sleeve gastrectomy fashioned with 60 mm stapler loads.
- Staple line reinforced with continuous 3-0 PDS suture.

Duodenal division
- Duodenum divided 6 cm from pylorus with 45 mm stapler.

Sympathectomy
- Selective dissection of two sympathetic fibers at right crus near cava.
- Circumferential 360° perivascular dissection of common hepatic artery just distal to gastroduodenal bifurcation.
- Aim: disrupt sympathetic input/output to hepatic region.

Ileal interposition
- Distal ileum divided 20 cm from ileocecal valve.
- 1.5 m ileal segment isolated and interposed.
- Side-to-side ileo-ileal anastomosis with stapler and Prolene closure.
- End-to-side duodeno-ileal hand-sewn anastomosis with 3-0 PDS.
- End-to-side ileo-jejunal anastomosis 50 cm from Treitz angle, mesenteric closure with Prolene.

Intra-operative outcomes

- Duration: 270 minutes (console 210).
- Blood loss: <100 mL.
- No intra-operative complications.

Postoperative course

- Early ambulation (6 hours).
- High-protein liquid diet after 12 hours, well tolerated.
- Discharged POD 4, no complications.
